# Automated Optically Guided System for Chemical Analysis of Single Plant and Algae Cells Using Laser Microdissection/Liquid Vortex Capture/Mass Spectrometry

**DOI:** 10.3389/fpls.2018.01211

**Published:** 2018-08-20

**Authors:** John F. Cahill, Vilmos Kertesz

**Affiliations:** Mass Spectrometry and Laser Spectroscopy Group, Chemical Sciences Division, Oak Ridge National Laboratory, Oak Ridge, TN, United States

**Keywords:** algae, single cell, mass spectrometry, liquid capture, laser microdissection, classification, high throughput, automated analysis

## Abstract

Current analytical methods are not capable of providing rapid, sensitive, and comprehensive chemical analysis of a wide range of cellular constitutes of single cells (e.g., lipids, metabolites, proteins, etc.) from dispersed cell suspensions and thin tissues. This capability is important for a number of critical applications, including discovery of cellular mechanisms for coping with chemical or environmental stress and cellular response to drug treatment, to name a few. Here we introduce an optically guided platform and methodology for rapid, automated recognition, sampling, and chemical analysis of surface confined individual cells utilizing a novel hybrid laser capture microdissection/liquid vortex capture/mass spectrometry system. The system enabled automated analysis of single cells by reliably detecting and sampling them either through laser ablation from a glass microscope slide or by cutting the entire cell out of a poly(ethylene naphthalate)-coated membrane substrate that the cellular sample is deposited on. Proof of principle experiments were performed using thin tissues of *Allium cepa* and cultured *Euglena gracilis* and *Phacus* cell suspensions as model systems for single cell analysis using the developed method. Reliable, hands-off laser ablation sampling coupled to liquid vortex capture/mass spectrometry analysis was conducted for hundreds of individual *Allium cepa* cells in connected tissue. In addition, more than 300 individual *Euglena gracilis* and *Phacus* cells were analyzed automatically and sampled using laser microdissection sampling with the same liquid vortex capture/mass spectrometry analysis system. Principal component analysis-linear discriminant analysis, applied to each mass spectral dataset, was used to determine the accuracy of differentiation of the different algae cell lines.

## Introduction

Cell-to-cell variation is inherent to multi-cellular organisms. Even starting from a single genetic precursor, natural stochastic cellular processes create variation in their chemistries over time (Chung et al., 2014). Knowledge of the chemical constituents (e.g., lipids, metabolites, proteins, etc.) in single cells is valuable for several important applications including determination of cellular function and specialization, understanding molecular mechanisms at the single cell level, and more (Zenobi, 2013; Comi et al., 2017). Despite this, most studies measure cellular chemistry in aggregate, and, thus, information on cellular variations is lost and understanding of cellular function impeded. Traditional methods for analyzing cellular makeup, including flow cytometry (Spitzer and Nolan, 2016) and immunohistochemistry (Walker et al., 2001), require manipulation of tissue which causes alteration of the chemical composition of the cell. In other cases, the used technique is specific for one or a small number of specifically targeted analytes (e.g., spectroscopic methods for metabolites such as ATP, NADH, etc.) (Papagiannakis et al., 2017). Nanomanipulation-based techniques manually extract cell contents, however, low throughput limits the value of the method as hundreds of cells need to be measured for a statistically sound analysis (Mizuno et al., 2008; Urban et al., 2010; Onjiko et al., 2017). Using laser ablation electrospray ionization-mass spectrometry (LAESI-MS), metabolic analysis was only achieved for major chemical constituents from 70 μm × 400 μm onion single cells (Shrestha and Vertes, 2009). Another MS-based technique, matrix assisted laser desorption/ionization-MS (MALDI-MS) has been used for single cell analysis from tissue (Li et al., 2000; Ong et al., 2015; Jansson et al., 2016) and, less commonly, from cellular suspensions (Urban et al., 2010). However, this method suffers from intense matrix signals in the <500 mass per charge range that may interfere with the signal of the analytes of interest (Zavalin et al., 2012). Thus, sensitive and rapid chemical analysis of individual cells of dispersed cell populations and of thin tissues remains a challenge that, if solved, could provide solutions for a number of important biomedical problems.

Laser microdissection-liquid vortex capture electrospray ionization-mass spectrometry (LMD-LVC/ESI-MS) is a recently developed ambient-ionization MS technique that combines the capabilities of LMD, microscopy, and MS through the use of a liquid-vortex capture probe for sample collection and transport to the ESI source (Cahill et al., 2015, 2016a,b, b2018). This technique has been shown to be highly sensitive relative to other ambient ionization-MS techniques, which has enabled sub-micrometer spatial resolution MS imaging capabilities (Cahill et al., 2015). In addition, chemical content of single cells was measured using a number of different sampling modes by LMD-LVC/ESI-MS (Cahill et al., 2016a). One of these sampling modes is “Cut and Drop” (CnD) sampling in which whole tissue microdissections are CnD using the laser from the microdissection system directly into the LVC probe (Cahill et al., 2016a). This mode of sampling has several advantages most notable of which is that it ensures 100% of sample material is transferred into the LVC probe, a feature lacking in most ambient-ionization techniques. This feature makes it the most sensitive mode of operation for LMD-LVC/ESI-MS. The advantages of CnD sampling were demonstrated by quantifying a drug (propranolol) present in hundreds of individual tissue microdissections from dosed animal tissues including mouse brain, liver, and kidney tissue (Cahill et al., 2016b) and by differentiating mouse brain tissue regions with high confidence without any additional sample preparation or workup (Cahill et al., 2018). In our previous work, cells of *Chlamydomonas reinhardtii*, ranging between 4 and 12 μm in diameter, were deposited on a glass substrate, air dried, and then sampled individually by laser ablation spot sampling (Cahill et al., 2016a). Two specific lipids [(18:3 and 16:4) monogalactosyldiacylglycerol (MGDG) and (18:4 and 16:0) diacylglyceryltrimethylhomo-Ser (DGTS)] were targeted and confidently identified in the individual cells. However, sampling of cells was accomplished manually, limiting the number of cells acquired and general applicability of the technique toward single cell analysis.

In this study, a novel automated optically guided system for chemical analysis of individual plant cells both for dispersed cell populations and for thin tissues is presented. A reliable cell recognition image analysis approach was developed and integrated with the current LMD-LVC-MS sample microdissection and analysis system. Once the cells were recognized, our in-house developed LMD support software generated appropriate laser beam paths based on the boundaries of the cells. This approach is similar to a recently introduced intelligent image-based *in situ* single-cell isolation system employing a different LMD system learning (Brasko et al., 2018). However, in the current system, the boundary information was used for either laser ablation of the entire content of the cell (thin tissue of *Allium cepa*) or for CnD sampling of cultured cells (*Euglena gracilis* and *Phacus*) for a subsequent online MS analysis. These latter cells were also used to classify with great confidence different plant cell lines using the chemical (mass spectrometric) information alone. The results reported here serve as the foundation for future studies using LMD-LVC/ESI-MS for robust, high throughput automated chemical analysis of single cells deposited on a surface.

## Materials and Methods

### Materials

LC-MS CHROMASOLV^®^ methanol + 0.1% formic acid (FA), chloroform, and water was purchased from Sigma-Aldrich (St. Louis, MO, United States). An *Allium cepa* (yellow onion) was purchased locally. The outer layers of epidermis cells were cut and placed on 1″ × 3″ glass microscope slides. *Euglena gracilis* and *Phacus* cells were purchased from Carolina Biological (Burlington, NC, United States). The commercial *Euglena gracilis* stock solution was diluted fourfold using water. The commercial *Phacus* solution was concentrated about 25-fold by first centrifuging 5 mL of stock cell solution at 1,500 RPM for 5 min using a centrifuge (Eppendorf 5430, Hauppauge, NY, United States) then removing the supernatant and resuspending the remaining pellet in 200 μL of water. An *Euglena gracilis*/*Phacus* cell mixture was created by mixing 50 μL of these treated (diluted and concentrated, respectively) cell solutions. Cells were deposited onto 4 μm polyethylene naphthalate (PEN) membrane slides (Leica Microsystems #11600289, Wetzel, Germany) by spotting 20 μL of the solution on the PEN slide and letting the sample air dry at room temperature.

### Chemical Analysis Using LMD-LVC/ESI-MS

The LMD-LVC/ESI-MS system has been described in detail in previous publications (Cahill et al., 2015, 2016a,b, 2018). Briefly, the system is comprised of a SCIEX TripleTOF^®^ 5600+ mass spectrometer (Sciex, Concord, ON, Canada) coupled to a Leica LMD7000 system (Leica Microsystems, Wetzel, Germany) via a low-profile LVC probe. The UV laser (349 nm, 5 kHz maximum repetition rate, and 120 μJ maximum pulse energy) in the LMD7000 system was used for laser raster sampling of individual epidermis cells of *Allium cepa* and CnD sampling of the cultured *Euglena gracilis* and *Phacus* algae cells. The LVC probe consists of a co-axial tube arrangement with a 1.12/1.62 mm (i.d./o.d.) outer stainless-steel probe and a 0.178/0.794 mm (i.d./o.d.) inner PEEK capillary. The probe was located 1 mm below the sample surface. Detrimental airflows near the probe were minimized by covering the LMD7000 with a plastic sheet and by attaching a sheath made of heat shrink tubing to the LVC probe that extended 1.1 mm above the top of the probe (∼0.1 mm from the sample surface). The LVC solvent flow rate was optimized at 100 μL/min 90/10% methanol/chloroform +0.1% FA to achieve a stable liquid vortex. Once in the solvent, analytes are extracted from the single cell and dissolved during transport to the ionization source of the mass spectrometer. The system is shown in **Supplementary Figure S1**.

The mass spectrometer was configured to acquire time-of-flight (TOF) mass spectra (mass/charge (*m/z*) 700–1000). Spectra were mass corrected based on a series of identified ions from known molecules present in each spectrum. A 0.05 s accumulation time, 5,500 V electrospray voltage, 100 V declustering potential, 400°C turbo heater temperature, and GS1 = 90 and GS2 = 60 N_2_ nebulizer gas settings were held constant across all experiments.

### Software Development

LMDCellCut was developed using Borland Delphi 7 computer language (Borland Software Corp., Scotts Valley, CA, United States) and can be run in any 32- or 64-bit Windows environment with at least 512 MB of RAM.

### Principal Component Analysis-Linear Discriminant Analysis (PCA-LDA) of Algae Cells

Principal component analysis-linear discriminant analysis was conducted using MATLAB^®^ (Mathworks, Natick, MA, United States).

## Results

### Automated Single Cell Recognition and Analysis

**FIGURE 1:**
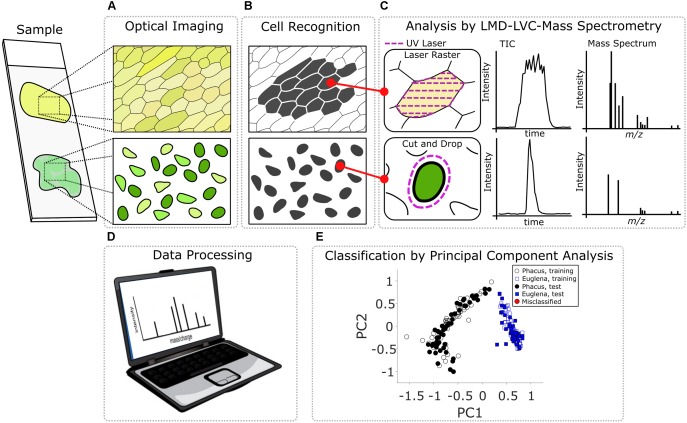
Steps of the optically guided cell selection, sampling, and analysis technology: **(A)** acquisition of the optical image of the sample; **(B)** application of image analysis algorithm for image segmentation and cell recognition; **(C)** sampling of selected cell and mass spectrometric data acquisition; **(D)** processing of mass spectrometric dataset; and **(E)** classification of analyzed cells.

The pipeline of the optically guided method for chemical analysis of plant single cells used in this study is shown schematically in **Figure 1**. In the first step, a glass microscope slide with *Allium cepa* tissue or a PEN slide with algae cells deposited on it (**Figure 1A**) was placed in the regular microscope slide holder of the LMD system. The in-house developed software *LMDCellCut* commanded the operating software of the LMD7000 to move to the upper left corner of the area to be examined. At that point, *LMDCellCut* obtained the optical microscope image of the sample (**Figure 1B**) by capturing the screen of the operating software of the LMD7000. The optical image was processed by an image analysis module (see section “**Supplementary Material**” for more details) of *LMDCellCut* that performed image segmentation (**Figure 1C**) and output individual cell boundary information. Using this information *LMDCellCut* directed the laser beam of the LMD to either raster the inside of the cell boundary (e.g., in case of *Allium cepa* tissue where spatially connected cells were analyzed, see **Figure 1C** top left panel) or to cut around the cell (e.g., in case of the algae cells where the cells are spatially distinct, see **Figure 1C** bottom left panel). It is important to note that the *LMDCellCut* software can change the field of view (see section “**Supplementary Material**” for more details) thus allowing the analysis of large areas of the microscope slide and hundreds of cells without any user intervention.

With laser raster sampling, cell particulates ejected as a result of laser ablation are sampled by the LVC probe, while in the case of CnD sampling the whole cell along with the PEN substrate was dropped into the LVC probe for a subsequent MS analysis (**Figure 1C** middle and right panels). Most importantly, manipulation of the mouse pointer and the mouse click events to control the laser in the operating software of the LMD were all accomplished automatically by the *LMDCellCut* software without any user intervention. After analysis, a separate in-house developed software package was used to extract mass spectra corresponding to a given cell (**Figure 1D**). Further mathematical processing of these spectra for PCA-LDA analysis and classification was accomplished using MATLAB^®^ (**Figure 1E**).

### Automated Analysis of Single *Allium cepa* Cells by Laser Ablation Raster Sampling

**FIGURE 2:**
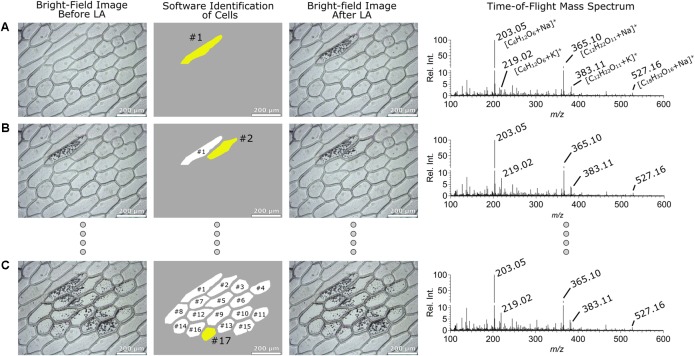
Bright-field optical images of the sampled *Allium cepa* epidermis tissue before (*column 1*) and (*column 3*) after sampling. The cell marked with *yellow in column 2* represents the cell analyzed (*white cells* indicates previously analyzed cells) by LA-LVC/ESI-MS analysis and *column 4* represents the corresponding positive ion mode ESI-MS TOF mass spectra. The sampled cells were #1, #2, and #17 in **(A–C)**, respectively.

*Allium cepa* cells are typically elliptical in shape and relatively large (∼150 μm). Thus, they are best sampled by rastering the laser inside their cell boundaries. In our earlier work (Cahill et al., 2016a) the *Allium cepa* cells were manually selected but in the present work we demonstrate the fully automated laser raster analysis of these cells. Column 1 in **Figure 2** shows optical images taken before sampling three respective *Allium cepa* cells indicated in column 2. The yellow shaded area represents the cell sampled in the given step, while the white areas indicate cells sampled in a previous step. In the particular case shown in **Figure 2**, the *LMDCellCut* software identified 17 cells that can be sampled in their entireties using the optical image of the non-sampled tissue. Subsequently, the boundary of an identified cell determined by the image analysis module of *LMDCellCut* (i.e., pixel coordinates on the optical image defining the contour of a cell) was drawn on the optical image in the LMD operating software. This was accomplished by first selecting the “Draw and Scan” option in the LMD operating software. It was followed by positioning the mouse pointer on the starting position of the cell contour and then by moving the mouse pointer with the left mouse button pushed down along the cell boundary. Once the entire shape is drawn, the left mouse button is released. Laser rastering commenced by clicking on the *Start* button in the LMD operating software. Note, that this kind of remote manipulation of the LMD laser system was partially employed in an earlier publication of ours for laser spot sampling and chemical imaging purposes (Lorenz et al., 2013). In the present case, the inside of the cell was targeted for laser ablation as to not disturb or influence adjacent cells with residual laser ablated material. Even if present, residual laser ablation material would constitute a negligible fraction of material relative to the cell. In these experiments, no evidence of cross-cellular contamination due to residual laser ablated material was observed. Column 3 in **Figure 2** shows the optical images (top to bottom) taken after laser rastering of cells #1, #2, and #17, respectively. The corresponding mass spectra collected during sampling are shown in column 4. These mass spectra were practically identical showing the high reproducibility of the current automated method. Briefly, the spectra were dominated by peaks corresponding to mono-, di-, and trisaccharides (marked in the figure) as expected on the basis of previous studies (Shrestha and Vertes, 2009; Cahill et al., 2016a). These peak assignments were verified by MS/MS analysis (data not shown) and previously reported ESI-MS spectra of these cells (Shrestha and Vertes, 2009).

### Automated Analysis of Single Algae Cells by Laser CnD Sampling

Automated monitoring of single *Chlamydomonas reinhardtii* cells (∼4–15 μm in diameter) for their MGDG and DGTS lipid content was demonstrated previously using the laser spot sampling mode (Cahill et al., 2016a). However, in that work a non-robust, very simple image analysis module was implemented that was unable to change the field of view of the optical image and would require several manual steps to acquire a large number of cells. In addition, the diameter of the laser spot was fixed that resulted in non-optimized sampling of cells with different sizes. Here we have improved on the single cell analysis method to enable automated analysis and the use of CnD sampling, which enables much greater sensitivity compared to laser ablation spot sampling due to more efficient capture of the sampled material.

**FIGURE 3:**
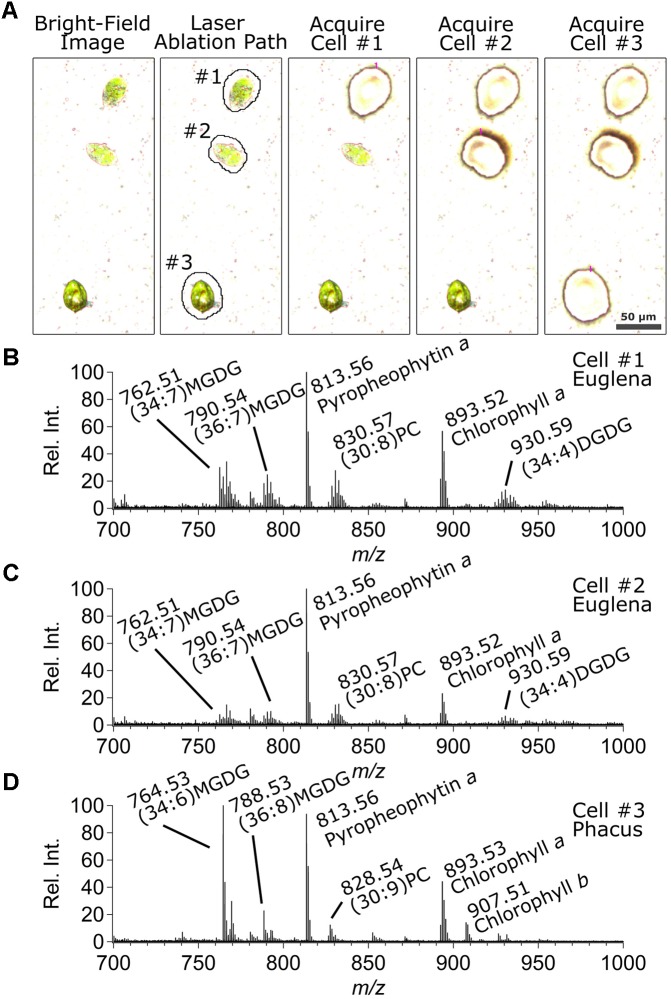
**(A)** Bright-field optical images of the sampled *Euglena gracilis* (*top* two cells, #1 and #2) and *Phacus* (*bottom* cell, #3) algae cells (*column 1*) before sampling, (*column 2*) showing the laser cut path derived by the image analysis algorithm, and after sampling cells (*column 3*) #1, (*column 4*) #2, and (*column 5*) #3. Positive ion mode ESI-MS TOF mass spectra corresponding to analyses of cells **(B)** #1, **(C)** #2, and **(D)** #3.

Columns 1–5 in **Figure 3A** show the original optical image, the optical image marked with the laser paths derived from the image analysis module, and optical images taken after sampling algae cells #1, #2, and #3, respectively. Preliminary experiments indicated that the cell wall of *Phacus* cells were strong enough to resist fracturing by osmotic effects once the cell is exposed to the solvent in the LVC probe. This in turn resulted in poor analyte extraction when doing CnD sampling. In contrast, *Euglena gracilis* and *Chlamydomonas reinhardtii* cells (Cahill et al., 2016b) were more susceptible to osmotic pressure-induced fracturing of the cell upon exposure to the solvent. In order to obtain the maximum MS signal regardless of cell identity, the cell wall was ruptured using a laser shot before CnD sampling. This was done in the *LMDCellCut* software by having it to select the “Move and Cut” option in the LMD operating software, followed by positioning the mouse pointer on the center of weight of the cell, and clicking on it causing the short laser pulse to puncture the cell. After that, the boundary of the selected area (i.e., pixel coordinates on the optical image defining the contour of an area larger than the cell, see section “**Supplementary Material**” for more details) determined by the *LMDCellCut* image analysis module was drawn on the optical image using the drawing tool of the LMD operating software. Specifically, this was accomplished by selecting the “Draw and Cut” option in the LMD operating software followed by moving the mouse pointer along the contour of the selected area, as described above for the *Allium cepa* cells. Laser CnD sampling was then commenced by clicking on the *Start* button in the LMD operating software which initiated laser ablation along the boundary of the selected area. This step could be repeated as many times as it is necessary to cut through the PEN membrane. Finally, the “Move and Cut” option in the LMD operating software was selected and the mouse pointer was positioned over the cell. A short click of the mouse then caused the firing of a laser pulse to eject the microdissected cell into the LVC probe. To ensure single cell analysis, only cells without neighboring cells within the boundary of the selected area were acquired. This prevents any problem of cross-contaminating signal by laser ablation of adjacent cells or influence of laser ablation on adjacent cells.

**Figures 3B–D** shows the corresponding mass spectra collected during CnD sampling of cells #1 (*Euglena gracilis)*, #2 (*Euglena gracilis)*, and #3 (*Phacus*), respectively. Mass spectra of *Euglena gracilis* cells in **Figures 3B,C** were similar and showed a different lipid distribution from that of *Phacus* in **Figure 3D**. MGDG and digalactosyldiacyl-glycerol (DGDG) lipids were observed as ammonium ([M + NH_4_]^+^) adducts which is common for ESI-MS analysis of these lipids (Han, 2016). Specifically, 34:7 and 36:7 MGDG, and 34:4 DGDG lipids were commonly observed in *Euglena gracilis* cells. In addition, significant peaks corresponding to 30:8 phosphatidylcholine (PC) and 34:4 DGDG were also observed. *Phacus* cells were rich in 34:6 and 36:8 MGDG, and 30:9 PC lipids. Peak assignments of these specific lipids were verified by MS/MS analysis (data not shown). In addition, mass spectra of both algae types showed the presence of the protonated forms of photosynthetic pigments chlorophyll *a*, chlorophyll *b*, and pyropheophytin *a*, though chlorophyll *b* was more strongly expressed in *Phacus* cells. Several other ions that suspected to be MGDG, DGDG, DGTS, and PC lipids were observed but not identified by tandem MS. These observations agreed in general with information about lipid and photosynthetic pigment content of alga cells (Sharma et al., 2015).

### PCA-LDA Differentiation of Algae Cell Types

One of the objectives of this work was to demonstrate the power of the automated LMD-LVC/ESI-MS single cell sampling and analysis approach with an emphasis toward enabling the discovery of statistical differences between cells via the chemical analysis of larger number of single cells with relative ease. Here we demonstrate the ability of automated LMD-LVC/ESI-MS analysis of 300 single algae cells and the use of PCA-LDA models of mass spectra to differentiate *Euglena gracilis* and *Phacus* cell lines.

**FIGURE 4:**
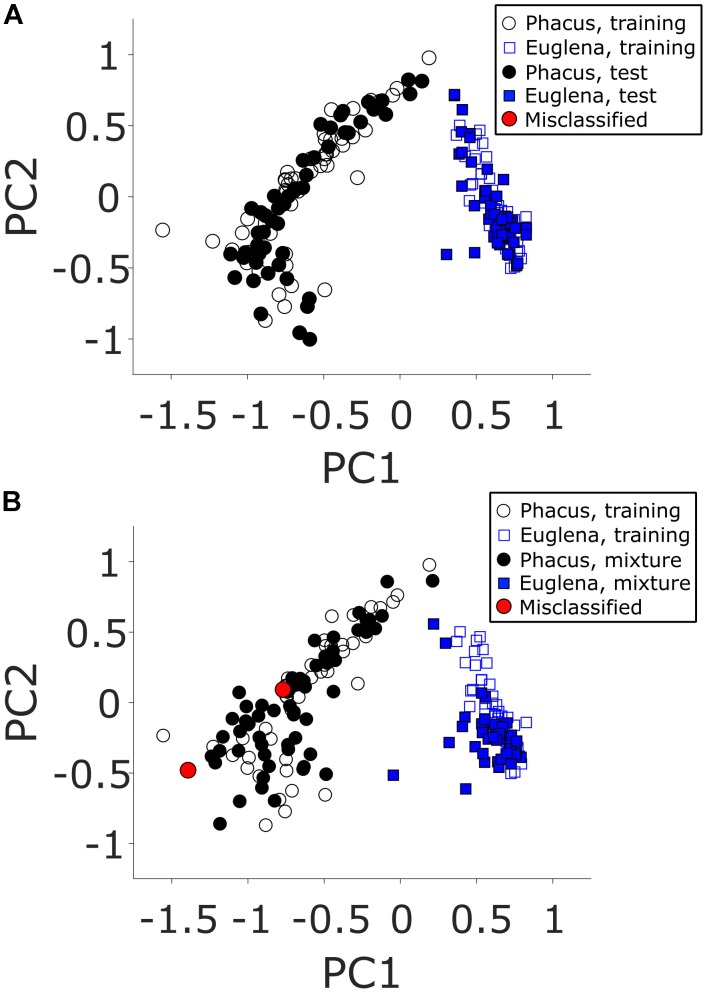
Two-component PCA models of positive mode ESI-MS TOF mass spectra for samples derived from analyses of **(A)** unmixed and **(B)** mixed (*blue squares*) *Euglena gracilis* and (*black circles*) *Phacus* algae cells. *Open circles* and *squares* represent the training samples (from analyses of unmixed cells in both panels) used to generate the PCA-LDA model. *Red circles* represent incorrectly classified test samples.

Principal component analysis-linear discriminant analysis training and test datasets were generated from sampling and analysis of 50 *Euglena gracilis* and 50 *Phacus* cells each (100 cells total). The cells were deposited on a PEN membrane slide from their respective cell solutions and left to air dry. Automated CnD sampling of individual cells was accomplished using the *LMDCellCut* software. Single cell mass spectra (*m/z* 700–1,000) were collected, normalized and binned into 0.1 *m/z* segments resulting in 3,000 data points per TOF mass spectrum. The top two principal components were used for model generation which captured 83.4% of sample variance (additional principle components did not significantly improve captured variance). The resulting PCA-LDA model created using the training and test datasets is shown in **Figure 4A**. Accuracy of this PCA-LDA model to correctly classify cell types using leave-one-out cross-validation was 100%.

To further test the system in a more realistic scenario, another dataset was generated using mass spectra of 100 additional single cells. These mass spectra were acquired from cells deposited from a mixed *Euglena gracilis/Phacus* cell solution. Manual identification of the 100 cells determined that 60 *Euglena gracilis* and 40 *Phacus* single cells were sampled and analyzed. The resulting PCA-LDA plot is shown in **Figure 4B**. The model fared well with only two miss-identified *Phacus* cells out of 100 total samples (98% apportionment accuracy). Manual inspection of the mass spectra of the incorrectly classified points (red circles) clearly indicated the samples originating from *Euglena gracilis* cells. As the manual identification has clearly indicated *Phacus* cells being sampled, the only reasonable explanation is that a previously microdissected *Euglena gracilis* cell was sampled together with a currently sampled *Phacus* cell. As the reliability of tissue microdissection capture is not 100% (Cahill et al., 2016b), it is theoretically possible that a previously cut *Euglena gracilis* cell was electrostatically attached to the surface and released when the *Phacus* cell was sampled. As demonstrated by the 2% apportionment error, this sampling error occurs infrequently and further modifications facilitating microdissection capture would reduce the likelihood of this error occurring in the future.

## Discussion

Herein, a novel automated optically guided system for chemical analysis of individual plant cells both for dispersed cell populations and for thin tissues with connected cells was demonstrated. We have developed a robust cell recognition image analysis approach that was integrated with the current LMD-LVC/ESI-MS sample microdissection and analysis system. Once the cells were recognized by our in-house developed *LMDCellCut* software, it generated appropriate laser beam paths based on the boundaries of the cells. This boundary information was used for either the laser raster sampling of the entire content of the cell when analyzing thin tissue of *Allium cepa* or for CnD sampling of spatially distinct *Euglena gracilis* and *Phacus* algae cells. *Euglena gracilis* and *Phacus* cells were differentiated with great confidence using only chemical (mass spectrometric) information.

In the present study, we demonstrated the system to be used with cells with circular (elliptical) shapes and with similar diameters in a given study (∼100 μm for *Allium cepa* tissue and ∼10 μm for the algae cells). To be more applicable for other (animal and human) cell types with irregular shapes on a wider size scale, detecting cell overlaps reliably, the cell recognition approach must be further improved. Means to improve the reliability of tissue microdissection capture are currently being investigated. In the analysis of the algae cell mixture 100 cells were acquired in ∼1 hr or ∼0.03 cell/s. Based on LVC/ESI-MS signal widths, throughput could be as fast as 1 cell/s but in the present experiments individual cells were often separated by great distances on the sample slide, which necessitated many movements of the LMD field-of-view decreasing sampling throughput. Increasing cell density on the PEN membrane slide surface would significantly help to improve sampling throughput. We did not focus on improving sampling throughput here, instead the focus was on demonstrating the completely automated cell finding, sampling, and analysis. Regardless, the results reported here serve as the foundation for future studies using LMD-LVC/ESI-MS for robust, automated chemical analysis of single cells deposited on a support surface.

## Data Availability

The datasets analyzed in the current study are available from the corresponding authors upon request. While the image processing algorithm is published in the current paper, the specific software code is not available due to pending copyright.

## Author Contributions

VK wrote in-house software LMDCellCut^TM^ for the image guided sampling. VK and JC designed the experiments and wrote the manuscript equally. JC executed the experiments, analyzed the data, and interpreted the results of all analysis. Both authors read and approved the final manuscript.

## Conflict of Interest Statement

The authors declare that the research was conducted in the absence of any commercial or financial relationships that could be construed as a potential conflict of interest.
