# Supplementary material for: Automated Optically Guided System for Chemical Analysis of Single Plant and Algae Cells Using Laser Microdissection/Liquid Vortex Capture/Mass Spectrometry
Source: Front Plant Sci. 2018 Aug 20;9:1211. doi: 10.3389/fpls.2018.01211 (PMC6110178; doi:10.3389/fpls.2018.01211)
Supplement: Supplementary file 1 [file Image_1.pdf]

Supplemental Information

Automated Optically-Guided System for Chemical Analysis of Single Plant and Algae Cells Using Laser Microdissection/Liquid Vortex Capture/Mass Spectrometry

John F. Cahill*,† and Vilmos Kertesz†

Mass Spectrometry and Laser Spectroscopy Group, Chemical Sciences Division, Oak Ridge National Laboratory, Oak Ridge, TN 37831-6131, USA
† J. F. C. and V. K. contributed equally to this work

*Corresponding Author:
John F. Cahill
Mass Spectrometry and Laser Spectroscopy Group
Chemical Sciences Division
Oak Ridge National Laboratory, Oak Ridge, TN 37831-6131
E-mail: cahilljf@ornl.gov
Phone: 865-574-4878

Running title: Automated Single Plant and Algae Cell Analysis

Submitted for consideration for publication in the special issue of ***Single Plant Cell Metabolomics"*** in ***Frontiers in Plant Science***

This manuscript has been authored by UT-Battelle, LLC under Contract No. DE-AC05-00OR22725 with the U.S. Department of Energy. The United States Government retains and the publisher, by accepting the article for publication, acknowledges that the United States Government retains a non-exclusive, paid-up, irrevocable, world-wide license to publish or reproduce the published form of this manuscript, or allow others to do so, for United States Government purposes. The Department of Energy will provide public access to these results of federally sponsored research in accordance with the DOE Public Access Plan (http://energy.gov/downloads/doe-public-access-plan).

Image Analysis Pipeline to Extract Contours of Connected Cells from an Optical Image of *Allium cepa* Epidermis Tissue. The image analysis pipeline is schematically shown in Figure S1. Once the color optical image of the tissue (Figure S1A) was obtained, it was converted into a grayscale image (Figure S1B). Several different grayscale conversion formulas were tested and the following formula resulted in the best segmentation of the *Allium cepa* epidermis tissue: the red/green/blue (R/G/B) components of the image were weighted using 0.3/0.59/0.11 (pixel intensity (PI)=0.3*R+0.59*G+0.11*B). This step was followed by correction of possible radial brightness gradient (it's abundance strongly depended on light conditions), i.e., a pixel in the center of the field of view is brighter than in the corners due to the use of a radial light source (Figure S1C). The image was then segmented using two-dimensional Otsu thresholding (Liu et al., 1993) resulting in Figure S1D. It was followed by noise filtering the segmented image by removing individual small "islands" of standalone segments which resulted in a "clean" segmented image schematically shown in Figure S1E. The segments corresponding to cells (represented by white "islands" in Figure S1E) was then filtered based on their size and if they touched any side of the image (i.e., the whole cell was not captured in the microscope image). Only whole cells with size above a given threshold were kept (colored cells in Figure S1F). Extracting the boundary pixels for each whole cell (shown in red in Figure S1G) was followed by application of a contour tracing algorithm (Liow, 1991) cell by cell to obtain the contour (i.e., a path through the pixels that defines the outer boundary of a cell) of all the cells (an example of a contour is shown schematically in Figure S1H). These contour lines were submitted to the operating software of the LMD7000 and used for rastering the inside of a cell (Figure S1I) during laser ablation of the onion cells.

Image Analysis Pipeline to Extract Contours of Cells from an Optical Image of Standalone Algae Cells. The image analysis pipeline is schematically shown in Figure S2. Steps in Figures S2A-S2E are identical to that of Figures S1A-S1E except that only the blue component of the color image in Figure S2A was used (PI= 1*B) when obtaining the grayscale image in Figure S2B. The segments corresponding to cells (represented by black "islands" in Figure S2E) was then filtered based on if they extended to any side

of the image (i.e., the whole cell was not captured in the microscope image). It was followed by extracting the boundary pixels for each whole cell (shown with black lines in Figure S2F). Contours of all cells were then determined using the same contour tracing algorithm as for the *Allium cepa* cells above (an example of a contour is shown schematically in Figure S2G). In the next step, the center of mass was determined for each cell (Figure S2H). In addition, extra space was added to the contour around the cell by moving a circle with a given radius along the contour (shown by the black line around the alga cell in Figure S2H) and using the outermost pixels to generate a new contour (indicated by the red line in Figure S2H) to ensure that a cell was sampled in its entirety. The center of mass locations and contour lines surrounding the cells were submitted to the operating software of the LMD7000. The center of mass information was used for positioning of laser shots slightly off-center of the cell before laser cutting (to puncture the cell) and to the center of the cell after laser cutting to eject the cell microdissection. The contour lines were used to guide laser cutting in the 'Cut and Drop' sampling of the algae cells (Figure S2I).

Automated Laser Control and Manipulation of the Operating Software of the LMD7000 from the *LMDCellCut* Software. The 'Move and Cut', 'Draw and Scan' and 'Draw and Cut' modes in the operating software of the LMD7000 were utilized to control the laser in the current work. These modes allow the user to fire the laser at a given location (used for laser spot sampling and utilized for rupturing the algae cells before 'Cut and Drop' sampling), to raster the laser inside a given shape (used for laser rastering and utilized for sampling onion cells) and to move the laser along the boundary of a given shape (used 'Cut and Drop' sampling of algae cells). Rather than manually controlling these modes, they were controlled by the in-house developed software package LMDCellCut©. Before an experiment was begun, the screen coordinates of the left uppermost corner, and width and height of the optical image was entered into the software in pixels. In addition, screen coordinates of several checkboxes (to select 'Move and Cut', 'Draw and Scan' and 'Draw and Cut' modes; to move the LVC probe in place (No Cap, Cap #4)) and buttons (to Start an experiment, to change the field of view, and to change the focus) were also specified. When actuated, LMDCellCut© mimicked an actual human user by positioning the mouse pointer at a specified location,

applying mouse movement and click event when needed.

Figure Captions:

Supplementary Figure S1. Schematic Image Analysis Pipeline for *Allium cepa* Epidermis Tissue. (A) Color, (B) grayscale and (C) radial brightness gradient-corrected images. Result of image segmentation (D) before and (E) after noise removal. (F) Whole cells with size above a given threshold and (G) their boundary pixels are shown in yellow and red, respectively. (H) Contour, i.e., the path that defines the outer boundary of a cell. (I) Laser rastering of the inside of the cell (red dashed lines) defined by the contour.

Supplementary Figure S2. Schematic Image Analysis Pipeline for Algae Cells. (A) Color, (B) grayscale and (C) radial brightness gradient-corrected images. Result of image segmentation (D) before and (E) after noise removal. (F) Boundary pixels of cells are shown in black. (G) Contour is shown by black line around green algae cell. (H) Center of mass and contour line with extra space around the cell shown by red dot and line, respectively. (I) Rupturing the cells by a (black filled circle) laser shot slightly off-center of the cells prior to the laser cut; (dashed red line) laser cut along the contour and (red filled circle) laser shot to the center of the cells after to the laser cut to eject/drop the cell into the LVC probe.

Supplementary Figures:

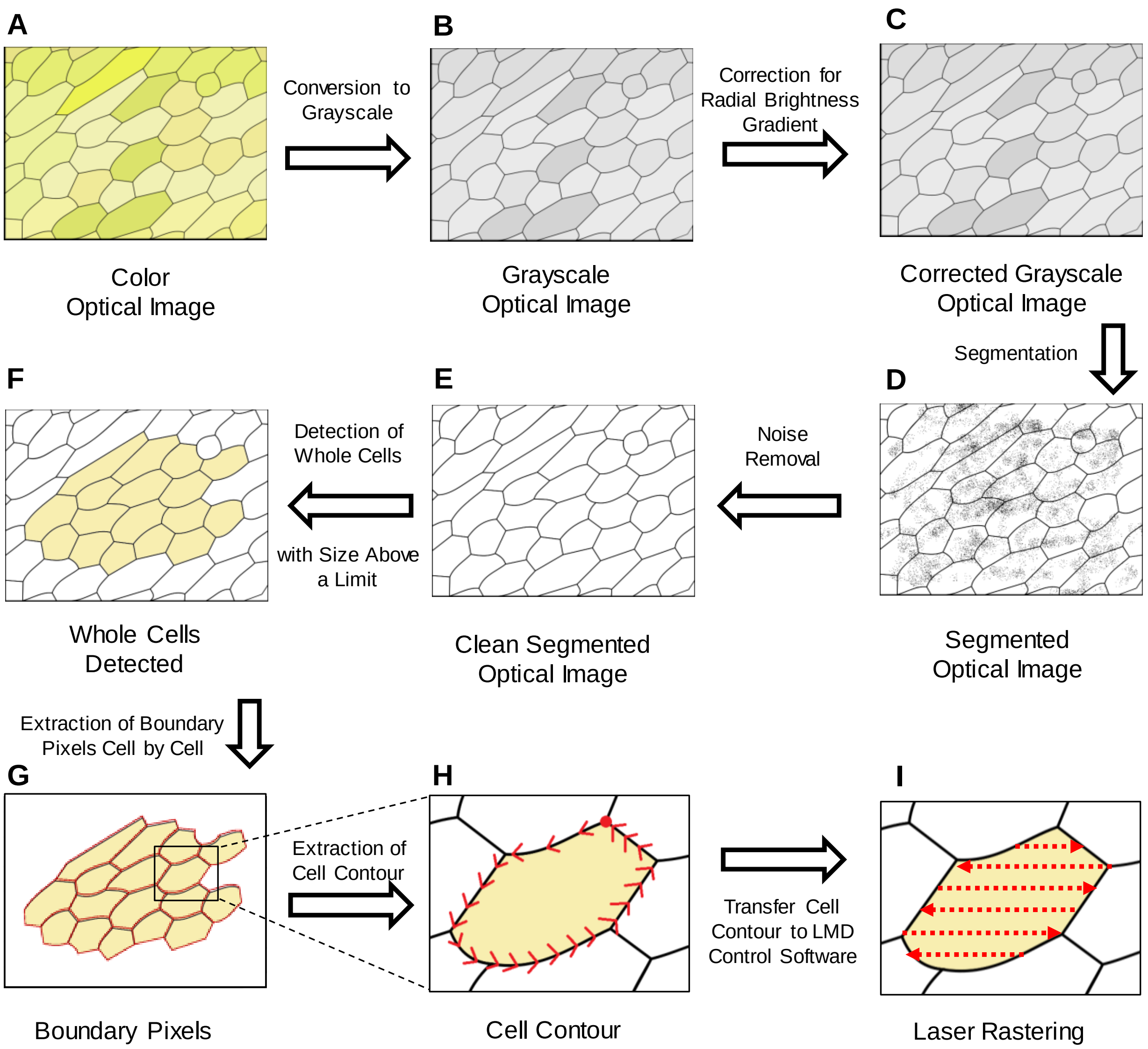

Figure S1.

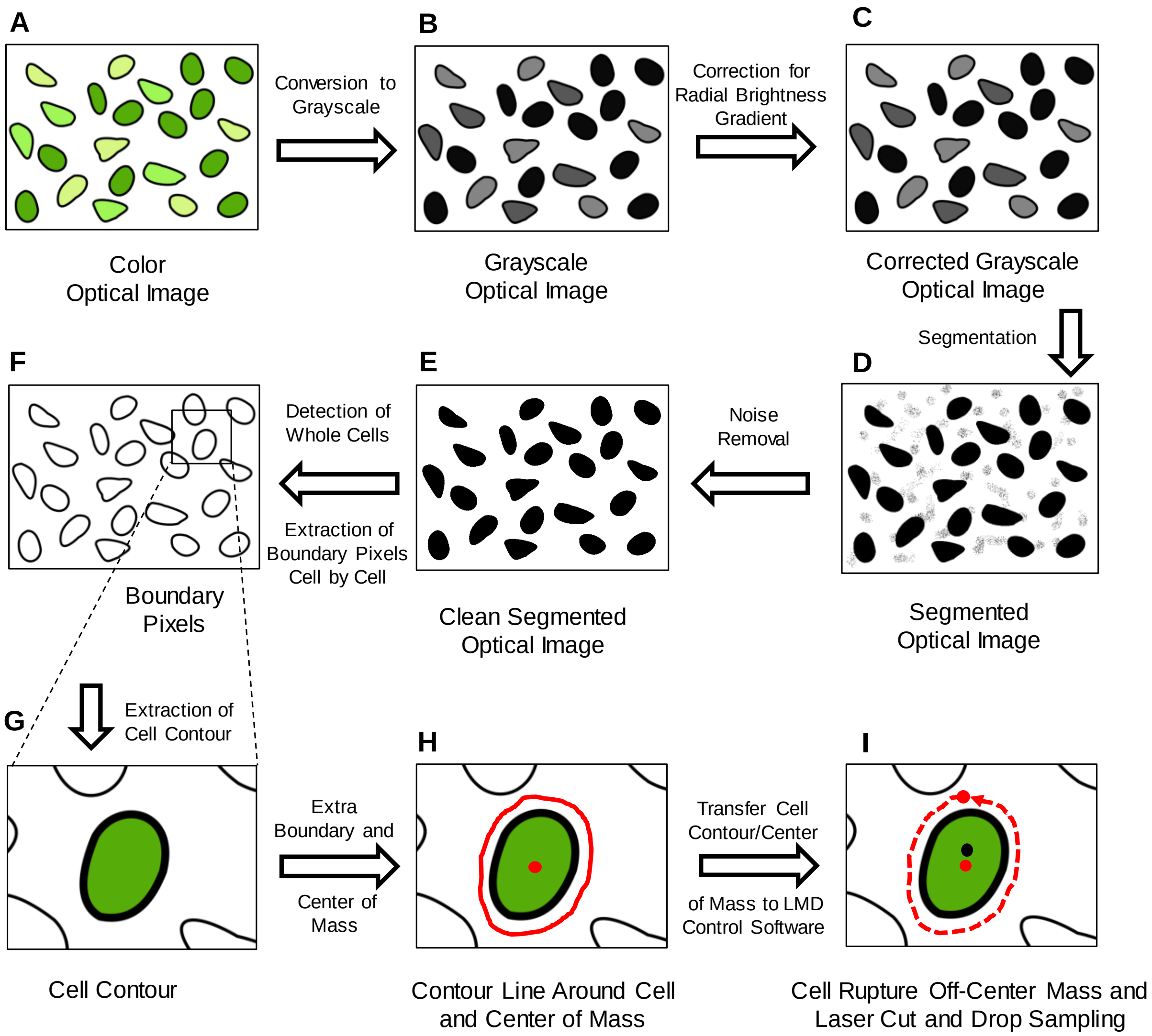
A
Conversion to Grayscale
B
Correction for Radial Brightness Gradient
C
Color Optical Image
Grayscale Optical Image
Corrected Grayscale Optical Image
Segmentation
F
E
D
Detection of Whole Cells
Extraction of Boundary Pixels Cell by Cell
Noise Removal
Boundary Pixels
Clean Segmented Optical Image
Segmented Optical Image
G
Extraction of Cell Contour
H
I
Extra Boundary and Center of Mass
Transfer Cell Contour/Center of Mass to LMD Control Software
Cell Contour
Contour Line Around Cell and Center of Mass
Cell Rupture Off-Center Mass and Laser Cut and Drop Sampling

Figure S2.

References:

J. Z. Liu, W. Q. Li. The automatic thresholding of gray-level picture via 2D Otsu method. Acta Automatica Sinica. 19(1): 101-105, 1993.

Y.-T. Liow. A contour tracing algorithm that preserves common boundaries between regions. CVGIP: Image Understanding
53(3), 1991, 313-321.
